# Diagnostic accuracy of AI-assisted *versus* independent physician interpretation for bone fractures: a systematic review and meta-analysis

**DOI:** 10.1186/s41747-026-00803-1

**Published:** 2026-09-17

**Authors:** Ming Zhang, Bihan Tang, Haoran Dai, Weiju Huang, Wangyang Bai, Jia He, Qi Chen

**Affiliations:** 1 School of Health Sciences and Engineering, University of Shanghai for Science and Technology, Shanghai, China; 2 Department of Health Statistics, Naval Medical University, Shanghai, China; 3 Department of Health Management, Naval Medical University, Shanghai, China; 4 Department of Radiology, The First Affiliated Hospital of Nanjing Medical University, Nanjing, China; 5 School of Medicine, Tongji University, Shanghai, China

**Keywords:** Artificial intelligence, Computer-assisted diagnosis, Deep learning, Fractures (bones), Meta-analysis

## Abstract

**Objective:**

We systematically evaluated the diagnostic performance of artificial intelligence (AI)-assisted interpretation *versus* independent physician assessment for fracture detection.

**Materials and methods:**

Adhering to PRISMA-DTA guidelines, we searched PubMed and Web of Science for original studies published up to September 17, 2025. Quality was assessed utilizing the QUADAS-3 framework. A bivariate random-effects model pooled diagnostic metrics. Accuracy was assessed by summary receiver operating characteristic (SROC) curves and its area under the curve (AUC). Mixed-effects meta-regression explored heterogeneity.

**Results:**

Of 450 records retrieved, after excluding 118 duplicates, the remaining 332 records were screened by title/abstract, identifying 38 candidates. Following full-text evaluation, 28 studies met the inclusion criteria, 17 of them suitable for meta-analysis. Compared to unassisted diagnosis, AI significantly improved pooled sensitivity (87%, 95% confidence interval [CI]: 84–89%, *versus* 73%, 95% CI: 69–78%) and maintained high specificity (95%, 95% CI: 92–97%, *versus* 94%, 95% CI: 89–96%). The SROC-AUC increased from 0.849 to 0.929. Subgroup analysis revealed junior clinicians derived the greatest benefit, exhibiting a 21% absolute sensitivity increase. Multivariate meta-regression, explaining 52.2% of heterogeneity, identified two-dimensional x-ray and junior physician status (*p* ≤ 0.005) as independent predictors of a lower absolute AI-assisted diagnostic ceiling.

**Conclusion:**

AI assistance is an effective diagnostic adjunct, substantially improving sensitivity and mitigating the experience gap for junior clinicians. However, multivariate evidence confirms AI cannot fully supersede the absolute diagnostic ceiling dictated by foundational clinical expertise and two-dimensional radiography’s physical limitations. Future workflows must optimize human-AI collaboration while maintaining a low threshold for cross-sectional imaging.

**Key Points:**

***Question*** Accurate fracture interpretation remains a significant challenge for non-specialists. This study quantifies how human-machine collaboration effectively mitigates clinical experience gaps and improves radiologic diagnosis.

***Findings*** AI assistance significantly increased overall pooled diagnostic sensitivity from 73% to 87% without compromising specificity, providing the greatest absolute diagnostic benefit to junior clinicians.

***Relevance Statement*** Integrating AI into high-pressure workflows substantially reduces missed fractures and safely bridges the experience gap for junior clinicians, ultimately optimizing patient triage and operational efficiency.

**Graphical Abstract:**

**Figure:**
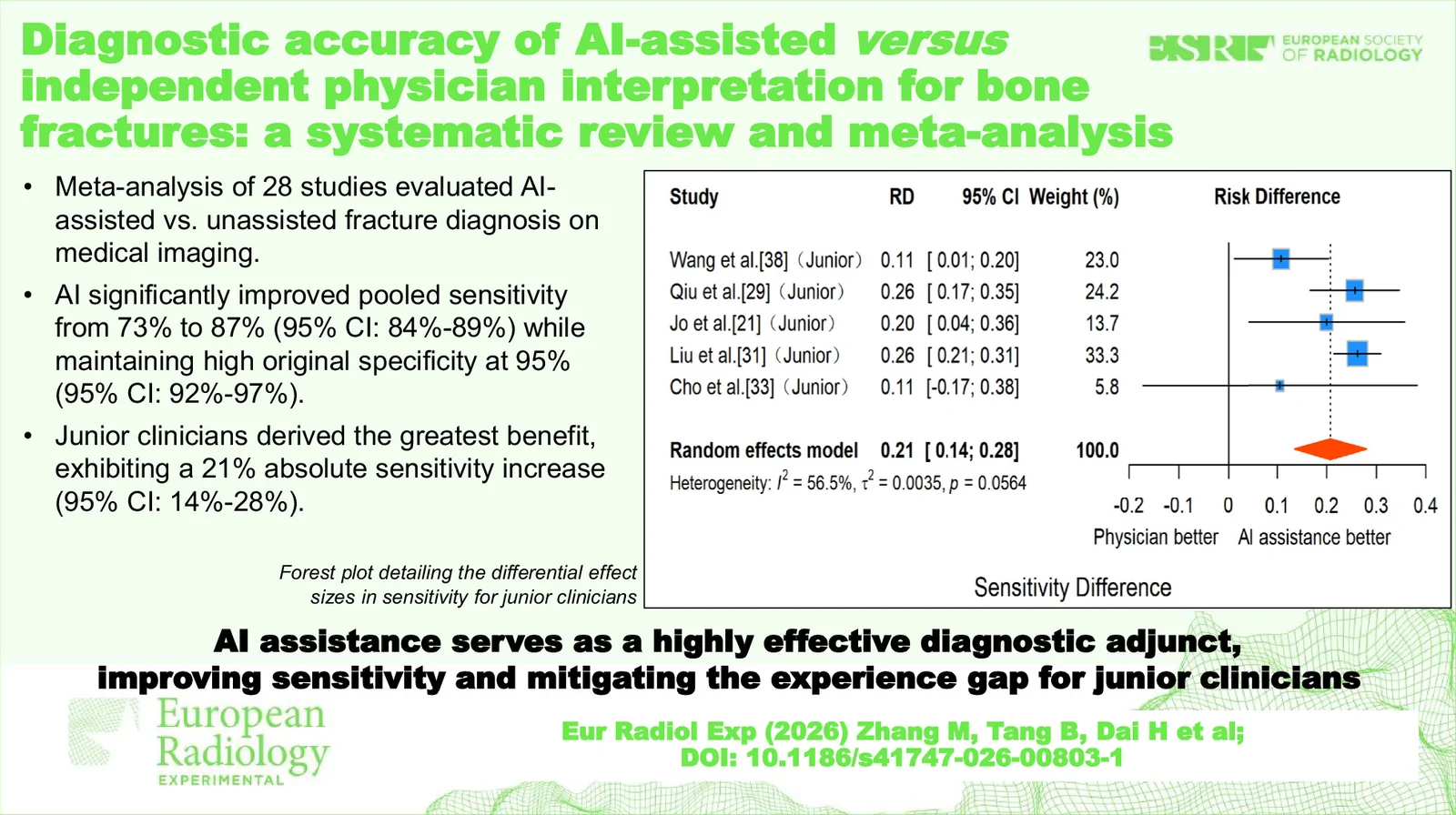

## Background

Bone fractures pose a substantial global health burden, with incidence rates reaching 1,229 per 100,000 person-years [1]. This demographic challenge is compounded by an aging population [2, 3]; United States hospitalization data demonstrate a consistent increase in fracture-related admissions, with over half (52–56%) occurring in patients aged 65 years or older [4]. While plain radiography remains the first-line screening modality due to its accessibility, it is inherently limited by anatomical superimposition, resulting in missed or delayed diagnoses in 1.9% to 7.6% of cases [5]. These diagnostic errors are frequently amplified by physician fatigue, high-volume workloads, and night-shift conditions in emergency departments [6–8], highlighting a critical need for reliable diagnostic adjuncts.

While deep learning algorithms demonstrate substantial potential to optimize diagnostic workflows [9, 10], the current literature remains fragmented. Early validation studies predominantly evaluated the standalone performance of algorithms against clinicians, failing to reflect the real-world clinical paradigm of “human-AI collaboration”. Furthermore, while initial evidence suggests AI may bridge the experience gap for junior clinicians [11–14], reported diagnostic accuracies vary widely. Crucially, the exact drivers of this inter-study variance—particularly the interplay between operator seniority and the inherent physical limitations of different imaging modalities (*e.g*., two-dimensional [2D] radiography *versus* three-dimensional (3D) cross-sectional imaging)—remain inadequately quantified.

Therefore, the objective of this study is to systematically review and meta-analyze the diagnostic accuracy of AI-assisted interpretation compared to independent physician assessment for bone fracture detection. Within this framework, “AI-assisted interpretation” is operationally defined as a collaborative diagnostic paradigm wherein deep learning algorithms function as clinical decision support tools—utilized via concurrent or second-reader workflows—to assist human clinicians rather than operating as autonomous diagnostic agents. Concurrently, “fracture detection” specifies the radiologic identification of structural discontinuity in osseous tissue across axial or appendicular skeletal structures visualized via conventional radiography, computed tomography, or magnetic resonance imaging, strictly excluding pathological fractures secondary to malignancies or image artifacts. Beyond pooling standard diagnostic metrics, we specifically utilized multivariate meta-regression to disentangle the confounding clinical variables driving inter-study heterogeneity. Ultimately, this work endeavors to provide high-quality, evidence-based guidance for the rational integration of AI into routine radiological workflows.

## Materials and methods

### Protocol and registration

This systematic review and meta-analysis was conducted in accordance with the Preferred Reporting Items for Systematic Reviews and Meta-Analyses (PRISMA) statement and its extension for Diagnostic Test Accuracy (PRISMA-DTA) guidelines [15, 16]. The study protocol was prospectively registered on PROSPERO (CRD420251035520).

### Search strategy and selection criteria

Two independent investigators (M.Z. and H.D., graduate researchers in medical imaging) searched the PubMed and Web of Science databases for original articles published between January 1, 2001, and September 17, 2025. The search strategy combined Medical Subject Headings (MeSH) and free-text terms, including “artificial intelligence”, “deep learning”, “radiologist”, “AI-assisted diagnosis”, and “fracture”. Detailed search strategies and PICOS (Population, Intervention, Comparison, Outcome, and Study design) criteria are provided in Supplementary Table S1.

Inclusion criteria were as follows: (1) original research published in English; (2) direct comparison of clinician diagnostic performance with *versus* without AI assistance for image-based fracture detection; and (3) provision of sufficient data to reconstruct 2 × 2 contingency tables (true positives, false positives, false negatives, and true negatives). Studies were excluded if they were: (1) reviews, conference papers, editorials, letters, case reports, or animal studies; (2) evaluations of standalone AI performance lacking a human comparator; or (3) based on inadequate reference standards.

### Data extraction and quality assessment

The same two investigators (M.Z. and H.D.) independently extracted data using a standardized template. Extracted variables encompassed study characteristics (first author, publication year, country, and study design), patient and imaging details (sample size, fracture type, and reference standard), AI algorithm specifications, and diagnostic performance metrics. The reconstructed 2 × 2 contingency table data for all included studies are detailed in Supplementary Table S2.

Methodological quality and applicability concerns were assessed independently using the recently updated QUADAS-3 tool [17]. Risk of bias was evaluated across four revised domains: Participants, Index Test, Target Condition, and Analysis. Assessments were conducted at the estimate level to capture differential biases between unassisted and AI-assisted workflows. Concerns regarding applicability were evaluated for the first three domains by mapping extracted data against a theoretically defined ideal test accuracy trial. Potential domain-level judgments included “Low”, “High”, or “Insufficient information”. Any discrepancies arising during literature screening, data extraction, or quality assessment were resolved through consensus or arbitration by a third senior reviewer (Q.C.), an associate professor with research expertise in medical imaging AI.

### Statistical analysis

When 2 × 2 contingency tables were not explicitly provided, true and false positives and negatives were derived from the reported sample sizes, disease prevalence, sensitivity, and specificity. A bivariate random effects model was used to calculate pooled sensitivity and specificity with 95% confidence intervals (CIs). Overall diagnostic accuracy was assessed by generating summary receiver operating characteristic (SROC) curves and calculating the area under the curve (AUC) [18]. Potential publication bias was evaluated using Deeks’ funnel plot asymmetry test, setting the significance threshold at *p* < 0.10.

Prespecified subgroup analyses were conducted based on physician seniority level, imaging modality, geographic region, and AI algorithm type. To ensure methodological transparency across heterogeneous study designs, a rigorous stratification protocol was applied for the seniority subgroup: (1) readers were categorized into “Junior” and “Senior” cohorts using a hierarchical definition: board-certified specialists were classified as “Senior” regardless of experience years; for non-certified clinicians, > 5 years of clinical practice designated “Senior” status, while ≤ 5 years defined the “Junior” cohort; (2) for studies providing explicitly decoupled data, cohorts were stratified accordingly; and (3) for mixed-cohort studies where AI-assisted interpretation was exclusively performed by a specific subgroup, only the corresponding unassisted cohort’s data were extracted to maintain a valid baseline comparison. Detailed cohort assignments and the extraction rationale for each included study are documented in Supplementary Table S3.

Bivariate mixed-effects meta-regression was performed to explore potential sources of inter-study heterogeneity. Evaluated covariates included clinical factors (publication year, imaging modality, physician seniority, and study design) and methodological quality indices derived from the QUADAS-3 assessment. For the latter, studies were dichotomized by risk of bias in the Participants domain (high risk [*e.g*., artificially enriched 1:1 prevalence] *versus* low risk [consecutive enrollment]) and the Target Condition domain (high risk [expert consensus based solely on 2D radiographs] *versus* low risk [computed tomography (CT)/magnetic resonance imaging (MRI) or longitudinal follow-up]). All statistical analyses were performed in R software (version 4.4.3) using the “mada” and “meta” packages, with a *p-*value < 0.05 considered indicative of a statistically significant moderator effect.

## Results

### Study selection and characteristics

**Fig. 1:**
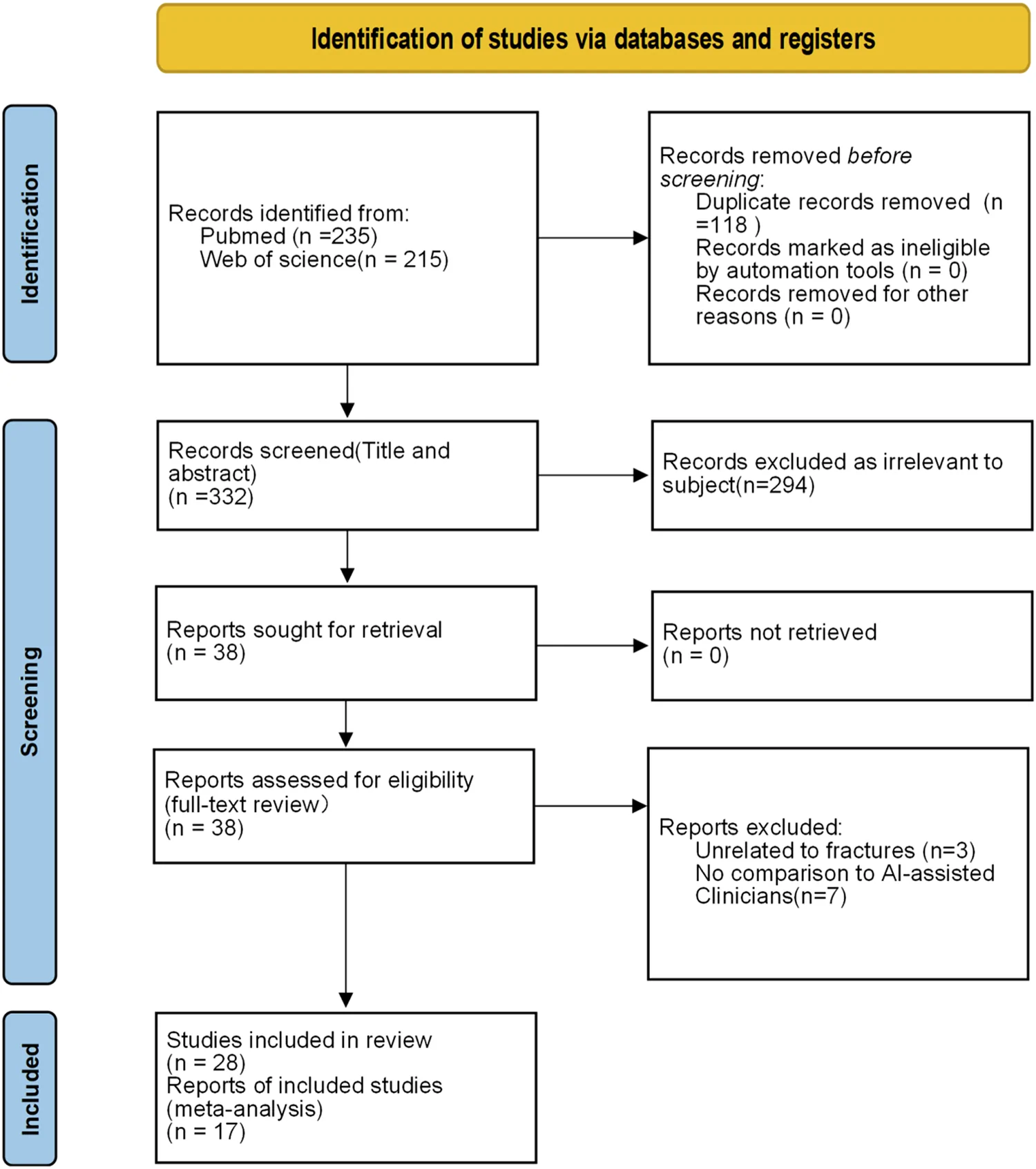
Flow diagram of the search and inclusion process

An initial literature search yielded 450 records. After excluding 118 duplicates, the remaining records were screened by title and abstract, identifying 38 candidate studies. Following full-text evaluation, 28 original studies [19–46] met the inclusion criteria for qualitative review. Of these, 17 studies provided complete 2 × 2 contingency data comparing independent physician assessments with AI-assisted diagnoses and were included in the quantitative meta-analysis (Fig. 1).

The 28 included studies were published between 2018 and 2025. The majority evaluated 2D plain radiographs (18 studies; [19, 25, 34]), while nine investigated CT [31, 38, 44] and one utilized MRI [21]. The assessed anatomical regions encompassed the appendicular skeleton, axial skeleton, and pediatric skull. Detailed study characteristics are summarized in Table 1. The evaluated AI interventions encompassed both regulatory-approved commercial platforms (*n* = 9)—predominantly Gleamer BoneView (*n* = 5)—and bespoke deep learning frameworks (*n* = 19). Architecturally, these customized models ranged from classical object detection networks (*e.g*., Faster R-CNN, YOLO variants) to advanced segmentation architectures (*e.g*., nnUNet, Attention U-Net). Their diagnostic outputs varied methodologically, delivering either regional bounding-box localization or high-fidelity pixel-level segmentation. Comprehensive technical specifications for all algorithms are detailed in Supplementary Table S4.

**Table 1:** Characteristics of the included studies

Study [reference number]	Year	Country	Study design	Imaging modality	Fracture type	Reference standard
Xing et al [19]	2024	China	Prospective	x-ray	Femoral neck fracture	2D radiograph consensus
Oppenheimer et al [20]	2023	Germany	Prospective	x-ray	Various fracture	2D radiograph consensus
Jaillat et al [22]	2025	France	Retrospective	x-ray	Pelvic and hip fractures	Clinical/radiologic follow-up
Meetschen et al [24]	2024	Germany	Retrospective	x-ray	Various fractures	2D radiograph consensus
Zech et al [25]	2024	United States	Prospective	x-ray	Fracture of the upper limb	2D radiograph consensus
Cohen et al [26]	2023	France	Retrospective	x-ray	Wrist fractures	Clinical/radiologic follow-up
Fu et al [27]	2023	United States	Retrospective	x-ray	Appendicular skeletal fractures	2D radiograph consensus
Duron et al [28]	2021	France	Retrospective	x-ray	Fractured bones in the limbs	2D radiograph consensus
Qiu et al [29]	2025	China	Retrospective	x-ray	Vertebral compression fracture	CT/MRI-based consensus
Yoon et al [30]	2023	United States	Prospective	x-ray	Scaphoid fracture	CT/MRI-based consensus
Lee et al [32]	2023	South Korea	Retrospective	x-ray	Wrist fractures	2D radiograph consensus
Choi et al [33]	2022	South Korea	Retrospective	x-ray	Skull fractures in children	CT/MRI-based consensus
Lindsey et al [34]	2018	United States	Retrospective	x-ray	Wrist fractures	2D radiograph consensus
Guermazi et al [36]	2022	United States	Retrospective	x-ray	Various fractures	2D radiograph consensus
Hendrix et al [37]	2023	Netherlands	Retrospective	x-ray	Scaphoid fracture	Clinical/radiologic follow-up
Yogendra et al [41]	2024	Singapore	Retrospective	x-ray	Pediatric appendicular Fractures	2D radiograph consensus
Keller et al [42]	2024	Switzerland	Prospective	x-ray	Distal radius fractures	CT/MRI-based consensus
Yuh et al [45]	2024	South Korea	Retrospective	x-ray	Thoracolumbar fractures	2D Radiograph consensus
Sun et al [23]	2025	China	Retrospective	CT	Rib fractures	Clinical/radiologic follow-up
Liu et al [31]	2021	China	Prospective	CT	Rib fractures	CT/MRI-based consensus
Zhou et al [35]	2023	United States	Retrospective	CT	Rib fractures	Clinical/radiologic follow-up
Wang et al [38]	2024	China	Retrospective	CT	Nasal bone fractures	CT/MRI-based consensus
Zhou QQ et al [39]	2019	China	Retrospective	CT	Rib fractures	CT/MRI-based consensus
Zhang et al [40]	2020	China	Retrospective	CT	Rib fractures	CT/MRI-based consensus
Inoue et al [43]	2022	Japan	Retrospective	CT	Fractures of pelvis, ribs and spine	CT/MRI-based consensus
Yao et al [44]	2021	China	Retrospective	CT	Rib fractures	CT/MRI-based consensus
Tan et al [46]	2023	China	Retrospective	CT	Rib fractures	CT/MRI-based consensus
Jo et al [21]	2023	South Korea	Retrospective	MRI	Thoracolumbar fracture	CT/MRI-based consensus

Regarding methodological quality, definitions of the target condition varied across the studies. Sixteen studies utilized 3D imaging (CT/MRI) or longitudinal clinical follow-up for the entire cohort [30, 33, 35] as the reference standard. The remaining studies primarily relied on 2D radiograph expert consensus or used cross-sectional imaging only for controversial cases [20, 28, 32], which was identified as a source of imperfect gold standard bias in our QUADAS-3 assessment.

The validation cohorts involved clinicians with varying expertise, ranging from junior residents to senior musculoskeletal radiologists. For studies reporting multiple reader performances, predefined representative data (*e.g*., pooled aggregate results or specific junior/senior subgroups) were extracted for the contingency tables to prevent statistical duplication.

### Quality assessment

Table 2 presents the methodological appraisal using the QUADAS-3 framework. In the Participants domain, a substantial proportion of studies exhibited a high risk of bias and high applicability concerns. This was primarily driven by the utilization of artificially enriched datasets (*e.g.*, test sets constructed with a 1:1 ratio of fractured to non-fractured radiographs) [25, 28, 36], which deviates from real-world clinical prevalence and introduces spectrum bias.

**Table 2:** Methodological analysis of the included studies based on the QUADAS-3 tool

Study [reference number]	Risk of bias				Applicability		
	Participants	Index test	Target condition	Analysis	Participants	Index test	Target condition
Xing et al [19]	High	Low	High	Low	High	Low	Low
Oppenheimer et al [20]	Low	Low	High	Low	Low	Low	Low
Jaillat et al [22]	Low	Low	Low	Low	Low	Low	Low
Meetschen et al [24]	High	Low	High	Low	High	Low	Low
Zech et al [25]	High	Low	High	Low	High	Low	Low
Cohen et al [26]	Low	Low	High	Low	Low	Low	Low
Fu et al [27]	High	Low	High	Low	High	Low	Low
Duron et al [28]	High	Low	High	Low	High	Low	Low
Qiu et al [29]	High	Low	Low	Low	High	Low	Low
Yoon et al [30]	High	Low	Low	Low	High	Low	Low
Lee et al [32]	Low	Low	High	Low	Low	Low	Low
Choi et al [33]	High	Low	Low	Low	High	Low	Low
Lindsey et al [34]	Low	Low	High	Low	Low	Low	Low
Guermazi et al [36]	High	Low	High	Low	High	Low	Low
Hendrix et al [37]	Low	Low	Low	Low	Low	Low	Low
Yogendra et al [41]	Low	Low	High	High	Low	Low	Low
Keller et al [42]	High	Low	Low	Low	High	Low	Low
Yuh et al [45]	Low	Low	High	Low	Low	Low	Low
Sun et al [23]	Low	Low	Low	Low	Low	Low	Low
Liu et al [31]	Low	Low	Low	Low	Low	Low	Low
Zhou et al [35]	High	Low	Low	Low	High	Low	Low
Wang et al [38]	High	Insufficient information	Low	Low	High	Insufficient information	Low
Zhou et al [39]	High	Low	Low	Low	High	Low	Low
Zhang et al [40]	Low	Low	Low	Low	Low	Low	Low
Inoue et al [43]	High	Low	Low	Low	High	Low	Low
Yao et al [44]	High	Low	Low	Low	High	Low	Low
Tan et al [46]	High	Low	Low	Low	High	Low	Low
Jo et al [21]	High	Low	Low	Low	High	Low	Low

Within the Target condition domain, studies utilizing 3D cross-sectional imaging (CT or MRI) or longitudinal clinical follow-up as the definitive ground truth were assessed as low risk [33, 39]. Conversely, studies relying exclusively on 2D radiograph expert consensus were classified as high risk, given the inherent limitation of 2D imaging in definitively ruling out occult fractures [19, 25, 34].

In the Index Test domain, the risk of bias was generally low, as most studies employed pre-specified operational thresholds and maintained appropriate blinding between the AI outputs and the unassisted human readers. Finally, in the Analysis domain, bias risks were primarily associated with inappropriate data handling or unit-of-analysis errors. For example, an exception to the generally low risk was noted in one study where verification bias was introduced by submitting only discordant cases (between human readers and the algorithm) for expert arbitration, rather than analyzing the entire enrolled cohort [41].

### Diagnostic performance

**Fig. 2:**
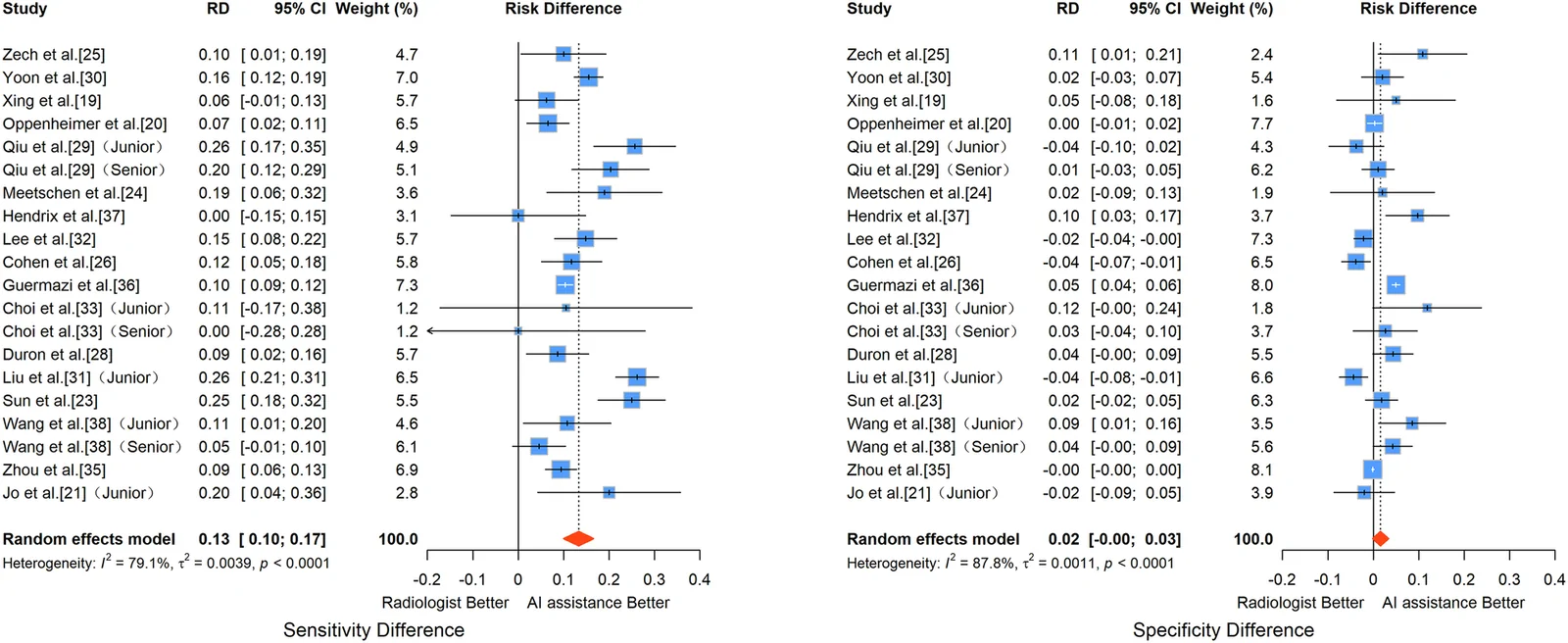
Forest plot detailing the differential effect sizes in sensitivity and specificity between AI-assisted physician diagnosis and unassisted physician diagnosis

Figure 2 illustrates the differential effect sizes in diagnostic sensitivity and specificity between AI-assisted and independent physician interpretations. Based on random-effects modeling, AI assistance yielded an absolute pooled sensitivity increase of 13% (95% CI: 10–17%). Sensitivity gains were observed in 18 of the 20 included datasets (90%), with Guermazi et al contributing the largest weight (10% increase; 95% CI: 9–12%). The junior cohorts evaluated by Qiu et al and Liu et al demonstrated sensitivity increases of 26% (95% CI: 17–35%) and 26% (95% CI: 21–31%), respectively. Pooled specificity exhibited an absolute increase of 2% (95% CI: 0–3%). Specificity levels were maintained or improved in 15 of the 20 datasets (75%). Significant inter-study heterogeneity was detected for both sensitivity (*I*² = 79.1%, *τ*² = 0.0039, *p* < 0.0001) and specificity (*I*² = 87.8%, *τ*² = 0.0011, *p* < 0.0001) differentials.

**Fig. 3:**
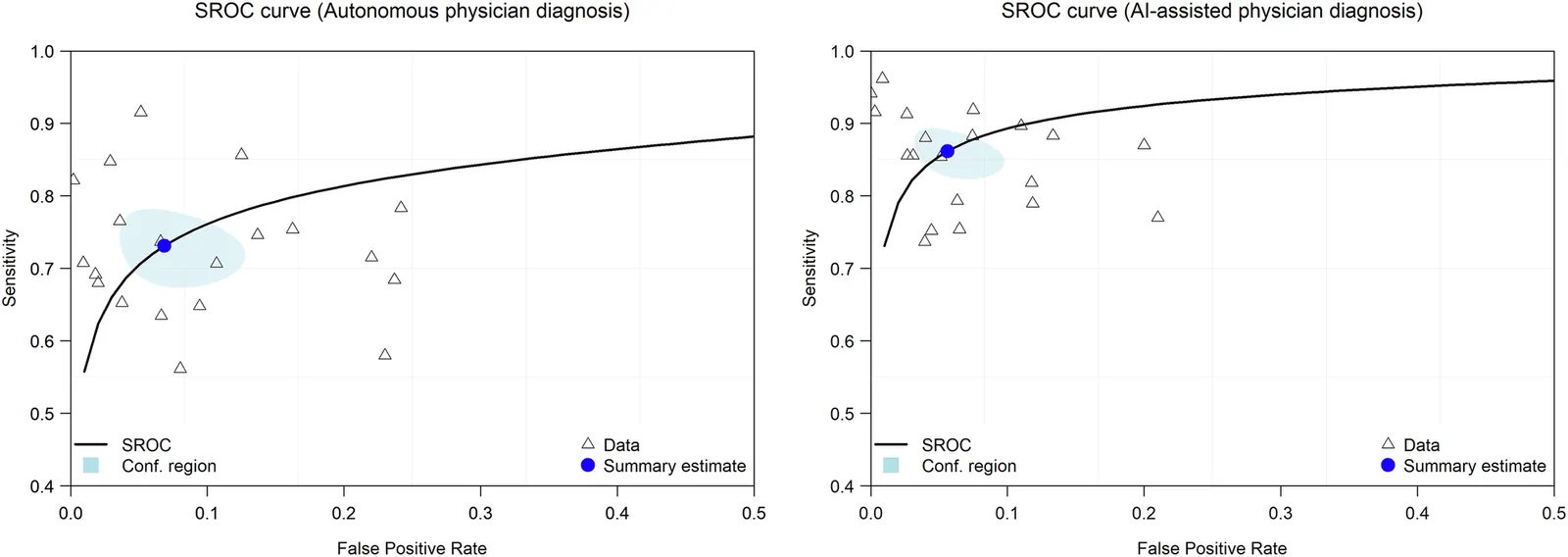
SROC curves comparing the overall diagnostic profiles of AI-assisted *versus* physician-alone interpretations

Overall diagnostic accuracy was compared using SROC curves (Fig. 3). Unassisted physician diagnosis yielded an AUC of 0.849, with a pooled sensitivity of 73% (95% CI: 69–78%) and specificity of 94% (95% CI: 89–96%). With the integration of AI assistance, the overall diagnostic performance improved, demonstrating an AUC of 0.929, with a pooled sensitivity of 87% (95% CI: 84–89%) and specificity of 95% (95% CI: 92–97%). Detailed forest plots for absolute pooled metrics are provided in Supplementary Fig. S9.

### Subgroup analysis

Subgroup analyses (Table 3 and Figs. S1–S8) confirmed that AI consistently prioritized sensitivity gains over specificity across all cohorts. Junior clinicians derived the most substantial benefit with a 21% (95% CI: 14–28%) sensitivity improvement, significantly exceeding the 10% (95% CI: -2 to 23%) gain in senior specialists (*p* < 0.05). Specificity improvements remained marginal for both junior 1% (95% CI: -5 to 7%) and senior 2% (95% CI: 0.1–5%) cohorts. Sensitivity increments were more pronounced in CT/MRI 16% (95% CI: 8–23%) *versus* x-ray 12% (95% CI: 9–15%), and in Asian centers 16% (95% CI: 11–22%) *versus* Western regions 11% (95% CI: 8–13%). Compared to Gleamer BoneView’s 10% (95% CI: 9–12%), bespoke algorithms yielded higher pooled sensitivity gains of 14% (95% CI: 10–19%); however, the former demonstrated superior stability with remarkably low heterogeneity (*I*^2^ = 13.7%, *p* = 0.3269) relative to other architectures (*I*^2^ = 80.3%, *p* < 0.0001).

**Table 3:** Subgroup analysis

Subgroup analysis	Sensitivity heterogeneity	Sensitivity improvement	Specificity heterogeneity	Specificity improvement
Junior doctor	*I*² = 56.5%, *τ*² = 0.0035, *p* = 0.0564	0.21 [0.14, 0.28]	*I*² = 74.0%, *τ*² = 0.0034, *p* = 0.0039	0.01 [-0.05, 0.07]
Senior doctor	*I*² = 78.7%, *τ*² = 0.0083, *p* = 0.0091	0.10 [-0.02, 0.23]	*I*² = 0.0%, *τ*² = 0, *p* = 0.5335	0.02 [0.001, 0.05]
CT or MRI	*I*² = 90.3%, *τ*² = 0.0076, *p* < 0.0001	0.16 [0.08, 0.23]	*I*² = 71.7%, *τ*² = 0.0011, *p* = 0.0034	0.01 [-0.02, 0.04]
X-ray	*I*² = 61.6%, *τ*² = 0.0019, *p* = 0.0013	0.12 [0.09, 0.15]	*I*² = 84.3%, *τ*² = 0.0012, *p* < 0.0001	0.02 [0.001, 0.04]
Europe and America	*I*² = 51.5%, *τ*² = 0.0006, *p* = 0.0357	0.11 [0.08, 0.13]	*I*² = 93.7%, *τ*² = 0.0012, *p* < 0.0001	0.02 [0.001, 0.05]
Asia	*I*² = 81.4%, *τ*² = 0.0060, *p* < 0.0001	0.16 [0.11, 0.22]	*I*² = 63.9%, *τ*² = 0.0010, *p* = 0.0020	0.01 [-0.02, 0.03]
Gleamer BoneView	*I*² = 13.7%, *τ*² < 0.0001, *p* = 0.3269	0.10 [0.09, 0.12]	*I*² = 91.3%, *τ*² = 0.0013, *p* < 0.0001	0.01 [-0.02, 0.05]
Other algorithms	*I*² = 80.3%, *τ*² = 0.0051, *p* < 0.0001	0.14 [0.10, 0.19]	*I*² = 65.9%, *τ*² < 0.0012, *p* = 0.0002	0.02 [-0.01, 0.04]

### Meta-regression and sources of heterogeneity

Bivariate mixed-effects meta-regression was conducted to evaluate sources of inter-study heterogeneity in AI-assisted diagnostic performance (Log diagnostic odds ratio, Log DOR) (Table 4). In univariate analysis, imaging modality was a significant moderator, with 2D x-ray associated with lower diagnostic efficacy compared to 3D CT/MRI (estimate: -1.83, 95% CI: -3.07, -0.60; *p* = 0.004). Physician seniority was not statistically significant in isolation (estimate: -1.52, 95% CI: -3.68, 0.63; *p* = 0.165). In the multivariate model adjusting for both imaging modality and physician seniority, 52.2% of the total inter-study heterogeneity was explained (*R*^2^ = 52.2%). In this adjusted model, both factors emerged as significant independent predictors. The use of 2D X-ray remained negatively associated with AI-assisted performance (estimate: -2.60, 95% CI: -3.79, -1.40; *p* < 0.001). Additionally, junior clinician status was independently associated with lower absolute AI-assisted performance compared to senior experts (estimate: -2.40, 95% CI: -4.08, -0.72; *p* = 0.005).

**Table 4:** Univariate and multivariate meta-regression analysis of potential sources of heterogeneity

Covariates	Univariate analysis		Multivariate model (*R*^2^ = 52.2%)	
	Estimate (95% CI)	*p*-value	Estimate (95% CI)	*p*-value
Imaging modality (x-ray *versus* CT/MRI^*^)	-1.83 (95% CI: -3.07, -0.60)	0.004	-2.60 (95% CI: -3.79, -1.40)	< 0.001
Physician seniority				
Junior *versus* senior^*^	-1.52 (95% CI: -3.68, 0.63)	0.165	-2.40 (95% CI: -4.08, -0.72)	0.005
Mixed *versus* senior^*^	-0.99 (95% CI: -2.91, 0.94)	0.315	-0.65 (95% CI: -2.11, 0.82)	0.387
Study design (prospective *versus* retrospective^*^)	0.28 (95% CI: -1.35, 1.71)	0.732	-	-
Publication year (continuous)	0.15 (95% CI: -0.48, 0.73)	0.614	-	-
ROB: participants (high *versus* low^*^)	0.48 (95% CI: -0.92, 1.88)	0.503	-	-
ROB: target condition (high *versus* low^*^)	0.51 (95% CI: -0.82, 1.84)	0.449	-	-

*ROB* Risk of bias

^*^ Reference category for the meta-regression model

### Publication bias

**Fig. 4:**
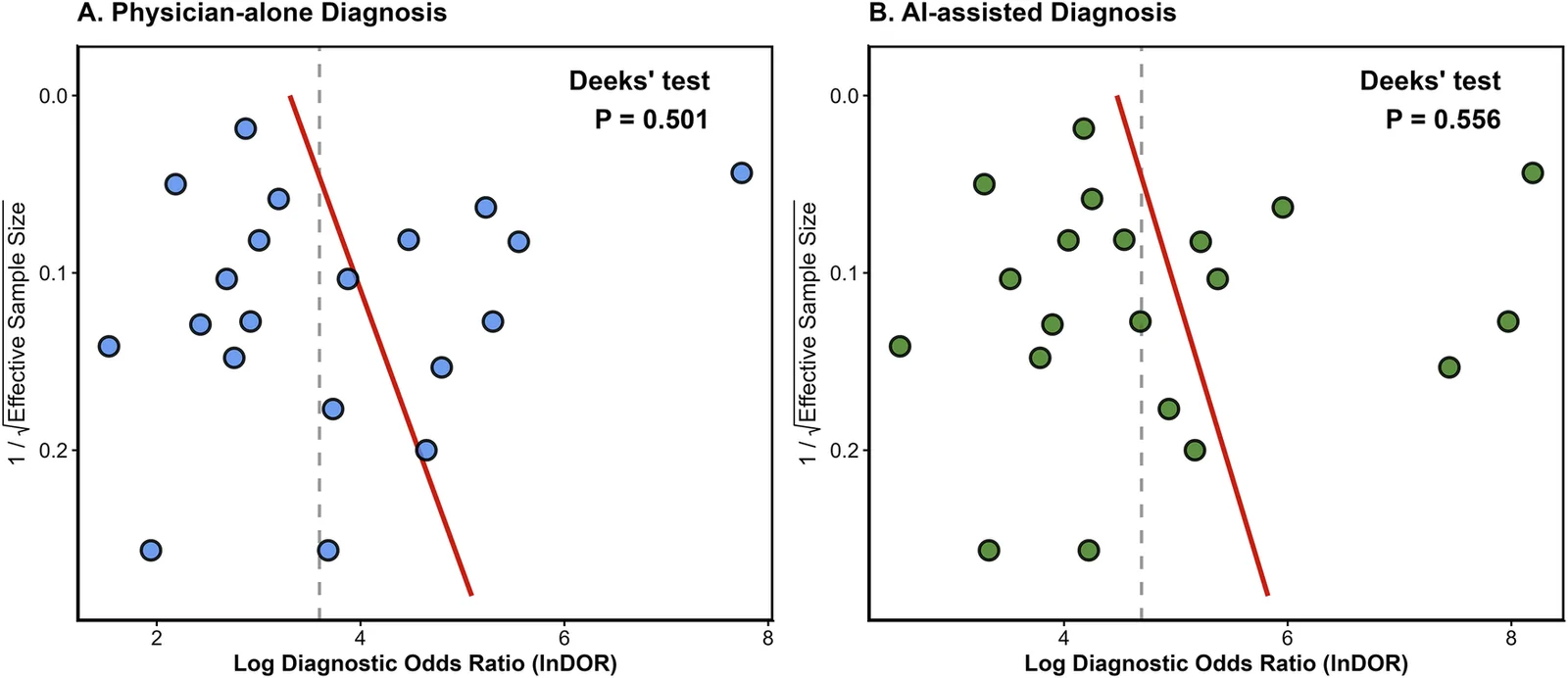
Deeks’ funnel plot asymmetry test for evaluating publication bias in (**A**) physician-alone diagnosis (*p* = 0.501) and (**B**) AI-assisted diagnosis (*p* = 0.556). The solid red line represents the regression line, and the dashed vertical line indicates the weighted mean of the log diagnostic odds ratio

Deeks’ funnel plot asymmetry test revealed no statistically significant asymmetry for either the physician-alone (*p* = 0.501) or AI-assisted (*p* = 0.556) assessments (Fig. 4), indicating a low risk of publication bias.

## Discussion

The integration of deep learning algorithms has increasingly demonstrated substantial potential to optimize fracture detection workflows. Rather than replacing clinicians, these tools serve as a reliable diagnostic adjunct to mitigate perceptual errors frequently induced by physician fatigue, experiential variance, and the inherent subtlety of occult lesions [47, 48]. By synthesizing 28 clinical investigations (incorporating 17 quantitative datasets), this study quantifies the incremental value of AI-assisted interpretation over independent physician assessment in real-world collaborative settings.

A primary finding of our meta-analysis is that AI-assisted workflows yielded a significant 13% absolute increase in pooled sensitivity (95% CI: 10–17%). This enhancement effectively stabilized the highly variable sensitivity baseline observed in unassisted interpretations (ranging from 46% to 93%) to a more consistent and narrower range of 68–99% with algorithmic support. Notably, while driving substantial gains in sensitivity, the AI-assisted modality preserved the original high specificity of clinicians (pooling at 95% compared to the baseline of 94%). This minimal diagnostic trade-off indicates that AI assistance does not merely lower the reporting threshold—which would inevitably inflate the false-positive rate—but fundamentally enhances the overall discriminative ability of the readers. Consequently, clinicians achieved a significant reduction in missed diagnoses (false negatives) without incurring a reciprocal penalty of increased misdiagnoses (false positives) [34, 36, 39, 43].

Beyond overall diagnostic accuracy, our findings demonstrate that AI effectively mitigates disparities in reader expertise, driving standardization across varying levels of training. Subgroup analysis confirmed that less experienced clinicians, including interns, junior radiologists, and emergency physicians, derived the most pronounced benefit, achieving a pooled sensitivity improvement of 21% (95% CI: 14–28%) [21, 22, 29, 31, 38]. In several cohorts, the diagnostic performance of junior physicians utilizing AI approached or even surpassed that of unassisted senior specialists [43, 44, 46]. Conversely, sensitivity gains for senior experts were typically marginal (< 5%) with confidence intervals crossing zero [24, 25, 27], indicating a ceiling effect where AI offers limited incremental utility for readers who already possess advanced pattern recognition skills. This underscores the potential of AI not simply as an automated reader, but as a complementary tool to accelerate the learning curve and bridge the experience gap in non-specialist interpreting pools.

Furthermore, the impact of AI on operational efficiency is substantial. Multiple included studies reported statistically significant reductions in image review time, with savings ranging from 2.6 to 51 s per case [36, 37, 39]. In high-pressure environments such as emergency departments (ED) and overnight shifts, where perceptual fatigue and high cognitive load frequently compromise reader accuracy, AI functions as a crucial diagnostic safety net. Within these settings, emergency physicians exhibited sensitivity increases of 14–24% [19, 22, 27, 40, 43]. Sun et al [23] corroborated this by demonstrating that AI-assisted radiologists achieved a patient-level sensitivity of 94.2% in trauma emergencies, representing a 25.0% improvement over traditional double-reading, while maintaining 100% specificity. These time-savings, coupled with pronounced sensitivity gains in acute settings, suggest that AI can alleviate emergency workflow bottlenecks without exacerbating the rate of false-positive referrals.

A critical strength of this study is the methodological appraisal. QUADAS-3 evaluation identified substantial structural flaws in several landmark validation studies: the construction of artificially balanced test sets with a 1:1 disease-to-normal ratio. This artificial enrichment deviates from true clinical prevalence, inherently introducing spectrum bias and artificially inflating pooled diagnostic metrics [25, 28, 36]. Furthermore, multivariate meta-regression was utilized to disentangle confounding clinical variables. This model revealed that imaging modality and physician seniority are independent statistical drivers, jointly explaining 52.2% of the observed heterogeneity. While subgroup analyses highlighted that junior clinicians derive the most pronounced incremental sensitivity gains from AI, the multivariate model underscores a crucial caveat: their absolute AI-assisted diagnostic ceiling remains significantly lower than that of senior experts (*p* = 0.005). This performance ceiling is also independently constrained by the imaging modality, with 2D X-ray exhibiting substantially lower diagnostic efficacy compared to 3D CT/MRI (*p* < 0.001). These statistical findings reflect two clinical realities. First, AI cannot entirely supersede the foundational anatomical intuition forged through years of clinical experience. Second, the physical limitation of anatomical superimposition in 2D radiography inherently restricts the algorithmic performance ceiling. In 2D imaging, AI functions primarily as a pattern-recognition enhancer for obscured cortical step-offs. Conversely, 3D cross-sectional modalities eliminate anatomical overlap, shifting the clinical challenge from visual ambiguity to perceptual fatigue caused by massive data volumes. Here, AI acts as an automated volumetric filter, mitigating observer fatigue rather than merely overcoming spatial ambiguity. Clinicians must therefore remain vigilant regarding the ceiling effects of both operator experience and 2D imaging, maintaining a low threshold for cross-sectional imaging when clinical suspicion persists.

While the aforementioned quantitative results define the current diagnostic baseline, a broader overview of the field highlights critical bottlenecks. To ensure generalized clinical robustness, multi-center validation across diverse hardware vendors serves as an important consideration for clinical deployment [49, 50]. Furthermore, algorithmic paradigms should evolve toward high-fidelity, explainable pixel-level segmentation utilizing advanced architectures [51, 52]. Future studies are strongly encouraged to report stringent spatial metrics (*e.g*., a minimum 0.8 Dice similarity coefficient) [53, 54] to validate algorithmic capability. Providing precise anatomical delineation through visual localization tools is essential for mitigating cognitive challenges like ‘automation bias’ and ‘algorithmic aversion’, thereby rebuilding clinical trust [55, 56]. Extrapolating from these diagnostic metrics to real-world implementation, prevailing perspectives in the literature emphasize that the field must pivot toward a symbiotic diagnostic ecosystem—a dynamic equilibrium where algorithms function as verifiable second readers to complement, rather than supersede, nuanced clinical intuition [57, 58].

Several limitations of this meta-analysis warrant consideration. First, the literature search was restricted to English-language publications, potentially excluding relevant international data. Second, the quantitative synthesis was constrained by the limited number of studies providing explicitly decoupled 2 × 2 contingency data for both unassisted and assisted readings. Third, the inclusion of primary studies with high risks of bias, specifically those utilizing artificially enriched 1:1 disease prevalence and imperfect 2D-only reference standards, may have upwardly skewed the pooled performance estimates. Fourth, architectural heterogeneity across evaluated AI platforms complicates direct comparisons. Although Gleamer BoneView was disproportionately represented, our subgroup analysis effectively mitigated single-vendor bias: bespoke and other commercial algorithms actually yielded higher pooled sensitivity gains than Gleamer (14% *versus* 10%). Nonetheless, future comparative studies remain necessary to confirm the generalizability of newly approved architectures. Fifth, the included literature lacks sufficient stratification for specific demographic subpopulations, particularly pediatric patients. Pediatric skeletal assessment presents unique anatomic complexities that often widen the diagnostic discrepancy between musculoskeletal specialists and general readers. Consequently, AI architectures trained predominantly on adult cohorts frequently demonstrate degraded diagnostic accuracy and limited generalizability when applied to pediatric cases [59].

In conclusion, AI-assisted interpretation serves as a highly effective diagnostic adjunct in bone fracture diagnosis, offering substantial improvements in sensitivity and operational efficiency, particularly for junior and emergency physicians. However, the inherent spatial limitations of 2D imaging remain a primary bottleneck. To advance the field, future research should ideally prioritize consecutive patient enrollment to avoid spectrum bias and utilize 3D cross-sectional imaging or longitudinal clinical follow-up as the definitive reference standard. Moreover, shifting towards advanced architectural pipelines capable of precise pixel-level delineation, validated by stringent spatial metrics, is critical to ensuring algorithmic reliability and fostering a clinically viable, evidence-based human-AI collaborative paradigm.

## Supplementary information

**Additional File 1: Table S1.** Literature search strategy and PICOS eligibility criteria. **Table S2.** Reconstructed 2×2 contingency tables for all included studies. **Table S3.** Physician seniority stratification: Rationale and individual study allocation. **Table S4.** Technical characteristics and validation strategies of included AI Models. **Figure S1.** Forest plots illustrating the effect of AI assistance on sensitivity and specificity among junior physicians. **Figure S2.** Forest plots illustrating the effect of AI assistance on sensitivity and specificity among senior physicians. **Figure S3.** Forest plots Illustrating the effect of AI assistance on sensitivity and specificity for X-Ray imaging. **Figure S4.** Forest plots illustrating the effect of AI assistance onsensitivity and specificity for CT or MRI modalities. **Figure S5.** Forest plots illustrating the effect of AI assistance onsensitivity and specificity in Asian cohorts. **Figure S6.** Forest plots illustrating the effect of AIassistance on sensitivity and specificity in European and American cohorts. **Figure S7.** Forest plots illustratingthe effect of AI assistance on sensitivity and specificity using the Gleamer BoneView algorithm. **Figure S8.** Forest plots illustrating the effect of AI assistance onsensitivity and specificity using other AI algorithms. **Figure S9.** Comparison of pooled absolute sensitivityand specificity between AI-assisted and independent physician diagnosis. (**a**) Pooled sensitivity for AI-assisted diagnosis; (**b**) Pooled specificity for AI-assisted diagnosis; (**c**) Pooled sensitivity for independent physician diagnosis; (**d**) Pooled specificity for independent physician diagnosis.

## Data Availability

All data generated or analyzed during this study are included in this published article and its supplementary information files.

## Abbreviations

2D: Two-dimensional
3D: Three-dimensional
AI: Artificial intelligence
AUC: Area under the SROC curve
CI: Confidence interval
CT: Computed tomography
MRI: Magnetic resonance imaging
PRISMA: Preferred Reporting Items for Systematic Reviews and Meta-Analyses
SROC: Summary receiver operating characteristic
