# Supplementary material for: Diagnostic accuracy of AI-assisted versus independent physician interpretation for bone fractures: a systematic review and meta-analysis
Source: Eur Radiol Exp. 2026 Sep 17;10:133. doi: 10.1186/s41747-026-00803-1 (PMC13586103; doi:10.1186/s41747-026-00803-1)
Supplement: Supplementary file 1 [file 41747_2026_803_MOESM1_ESM.pdf]

Diagnostic accuracy of AI-assisted versus independent physician interpretation for bone fractures: a systematic review and meta-analysis

ELECTRONIC SUPPLEMENTARY MATERIAL

Table S1 Literature Search Strategy and PICOS Eligibility Criteria

Acronym	Search strategy
P(Population)	Patients requiring fracture diagnosis
I(Intervention)	AI-assisted diagnosis (algorithm/software support)
C(Comparison)	Physician-alone diagnosis (without AI assistance)
O(Outcomes)	Diagnostic accuracy, sensitivity, specificity, AUC
S(Study type)	Original research studies

Detailed Database Search Strings

1. PubMed Search Strategy

((("deep learning" OR "artificial intelligence" OR "AI" OR "convolutional neural network*")
AND ("radiologist*" OR "clinician*" OR "physician*")
AND ("fracture*" OR "bone injury" OR "fracture detection"))
AND ("comparison" OR "versus" OR "with and without" OR "collaboration" OR "assist*"))
NOT (animal* OR rat OR mouse)

2. Web of Science Search Strategy

(TS=("deep learning" OR "artificial intelligence" OR "AI" OR "convolutional neural network*"))
AND (TS=("radiologist*" OR "clinician*" OR "physician*"))
AND (TS=("fracture*" OR "bone injury" OR "fracture detection"))
AND (TS=("comparison" OR "versus" OR "with and without" OR "collaboration" OR "assist*"))
NOT (TS=("animal*" OR "rat" OR "mouse"))

Table S2. Reconstructed 2×2 contingency tables for all included studies

Author	doctor_TP	doctor_FP	doctor_TN	doctor_FN	AI_assisted_TP	AI_assisted_FP	AI_assisted_TN	AI_assisted_FN
Zech et al. [25]	94	29	91	26	106	16	104	14
Yoon et al. [30]	858	132	468	342	1044	120	480	156
Xing et al. [19]	137	5	35	23	147	3	37	13
Oppenheimer et al. [20]	311	23	773	56	335	21	775	32
Qiu et al. [29] (Junior）	105	15	172	82	153	22	165	34
Qiu et al. [29] (Senior）	122	7	180	65	160	5	182	27
Meetschen et al. [24]	58	23	77	42	77	21	79	23
Hendrix et al. [37]	49	25	129	16	49	10	144	16
Lee et al. [32]	191	3	320	79	231	10	313	39
Cohen et al. [26]	189	14	376	58	218	29	361	29
Guermazi et al. [36]	3732	543	5217	2028	4331	256	5504	1429
Choi et al. [33] (Junior)	13	18	58	6	15	9	67	4
Choi et al. [33] (Senior)	14	5	71	5	14	3	73	5
Duron et al. [28]	212	32	268	88	238	19	281	62
Liu et al. [31] (junior)	356	41	577	205	503	68	550	58
Sun et al. [23]	130	1	54	58	177	0	55	11
Wang et al. [38] (Junior)	97	16	101	33	111	6	111	19
Wang et al. [38] (Senior)	119	6	111	11	125	1	116	5
Zhou et al. [35]	585	4	1996	127	652	6	1994	60
Jo et al. [21] (Junior)	34	1	49	16	44	2	48	6

Table S3. Physician Seniority Stratification: Rationale and Individual Study Allocation

Study (Ref.)	Reader Composition & Clinical Experience	Junior Cohort Allocation	Senior Cohort Allocation	Extraction Strategy & Methodological Rationale
Qiu et al. [29]	4 radiologists & 1 spine surgeon:<br>(1) Readers 1, 2, 5: 3–5 yrs<br>(2) Readers 3, 4: 10–15 yrs	Readers 1, 2, 5	Readers 3, 4	Stratified based on the 5-year clinical experience threshold. Individual reader data were explicitly decoupled in the original report.
Choi et al. [33]	5 observers:<br>(1) radiology residents (2 yrs)<br>(2) emergency physicians (5 yrs)<br>(3) pediatric radiologist (8 yrs)	Residents & ED physicians	Pediatric radiologist	ED physicians and residents were pooled into the Junior cohort as they met the ≤ 5 years criterion. The 8-year specialist was assigned to the Senior cohort.
Liu et al. [31]	6 junior radiologists (approx. 2 years of trauma imaging experience)	All 6 readers	None	The study exclusively evaluated junior clinicians; thus, the total aggregate performance was allocated to the Junior subgroup.
Wang et al. [38]	2 radiologists:<br>(1) 1 low-seniority (limited experience)<br>(2) 1 high-seniority (highly experienced)	Low-seniority reader	High-seniority reader	Stratified by the original study's predefined seniority levels. Separate performance data for both clinicians were extracted.
Jo et al. [21]	2 radiologists:<br>(1) Reader 1: MSK specialist (10 yrs)<br>(2) Reader 2: Resident (3 yrs)	Reader 2	Excluded	Baseline Comparability Protocol: Reader 1 was excluded as they only performed manual segmentation and did not participate in AI-assisted assessment, precluding a valid baseline comparison.

Table S4. Technical Characteristics and Validation Strategies of Included AI Models

Study	AI algorithm or Method	Validation(internal, external)	Diagnostic result(AI)
Zech et al. [25]	Faster R-CNN X101-FPN	I: Yes E: Yes	AUC: 0.892, Se: 90.0%, Sp: 88.3%
Yoon et al. [30]	CNN	I: Yes E: Yes	Se: 79%, Sp: 72%
Xing et al. [19]	Faster R-CNN,DenseNet-121,U-Net	I: Yes E: No	Acc: 94.1%, AUC: Garden I/II 0.94, Garden III/IV 0.99
Oppenheimer et al. [20]	Gleamer BoneView (Detectron 2)	I: Yes E: No	Se: 86.92%, Sp: 84.67%
Keller et al. [42]	CNN	I: Yes E: No	Acc: 98.5%, AUC: 0.995, Se: 98%, Sp: 98%
Qiu et al. [29]	nnUNet,ResNet18	I: Yes E: Yes	AUC: 0.942, Se: 78.4%, Sp: 90.9%
Jaillat et al. [22]	Gleamer BoneView (Detectron 2)	I: Yes E: Yes	Pelvis: Se:81.04% ; Hip: Se:96.76%
Meetschen et al. [24]	Gleamer BoneView (Detectron 2)	I: Yes E: Yes	Se: 93%, Sp: 77%
Yogendra et al. [41]	Radiobotics RBfracture V.2	I: Yes E: No	Acc: 97%, Se: 97%, Sp: 97%
Yuh et al. [45]	Mask R-CNN (DEEP:SPINE)	I: Yes E: Yes	ICC: 0.94
Hendrix et al. [37]	CNN	I: Yes E: Yes	Se: 72%, Sp: 93%
Lee et al. [32]	RetinaNet,DeepLab v3,NasNet	I: Yes E: No	Se: 97.7%, Sp: 93%
Cohen et al. [26]	Gleamer BoneView (Detectron 2)	I: Yes E: Yes	Se: 83%, Sp: 96%
Fu et al. [27]	Rayvolve	I: Yes E: Yes	Acc: 98%, Se: 98%, Sp: 88%
Guermazi et al. [36]	Detectron 2 optimized model	I: Yes E: Yes	AUC: 0.97
Choi et al. [33]	YOLOv3	I: Yes E: Yes	AUC: 0.922, Se: 81.1%, Sp: 91.3%
Duron et al. [28]	Gleamer BoneView (Detectron 2)	I: Yes E: Yes	AUC: 0.94
Lindsey et al. [34]	CNN	I: Yes E: No	AUC: 0.975, Se: 93.9%, Sp: 94.5%
Liu et al. [31]	RB-V-Net	I: Yes E: Yes	NA
Sun et al. [23]	uAI-BoneCare	I: Yes E: No	Se: 95.7%, Sp: 94.7%
Wang et al. [38]	YOLOX,GhostNetv2	I: Yes E: Yes	Acc: 97.44%, Se: 95%, Sp: 95%

Tan et al. [46]	InferRead CT Bone (CNN)	I: Yes E: No	NA
Zhou et al. [35]	U-Net,Faster R-CNN,ResNet50	I: Yes E: Yes	Se: 79.35%, Sp: 84.9%
Inoue et al. [43]	Faster R-CNN	I: Yes E: No	Acc: 64.8%, Se: 78.6%
Yao et al. [44]	U-Net,DenseNet	I: Yes E: No	Acc: 0.869, Recall: 0.913
Zhang et al. [40]	Faster R-CNN	I: Yes E: No	Se: 79.4%
Zhou QQet al. [39]	Faster R-CNN	I: Yes E: Yes	F1-score: 0.849
Jo et al. [21]	Attention U-net,Inception-ResNet-V2	I: Yes E: Yes	AUC: 0.928, Se: 88%, Sp: 82%

Note: Se = Sensitivity; Sp = Specificity; Acc = Accuracy; AUC = Area Under the Curve; I = Internal validation; E = External validation

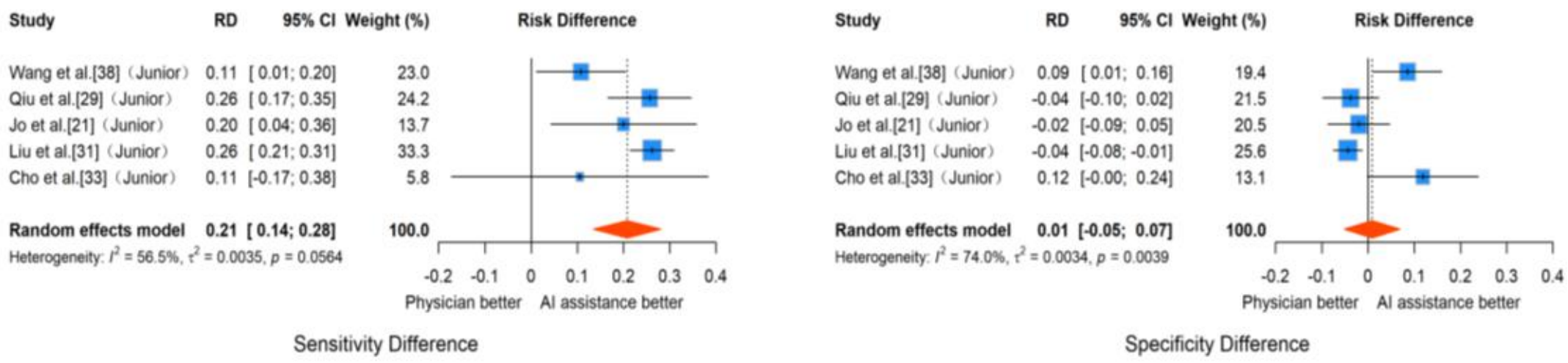

Figure S1. Forest Plots Illustrating the Effect of AI Assistance on Sensitivity and Specificity Among Junior Physicians

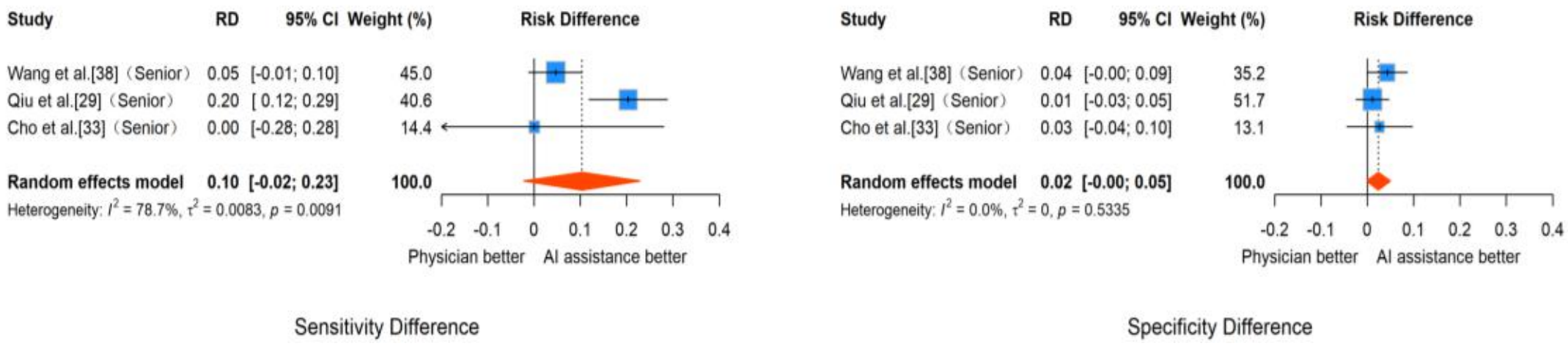

Figure S2. Forest Plots Illustrating the Effect of AI Assistance on Sensitivity and Specificity Among Senior Physicians

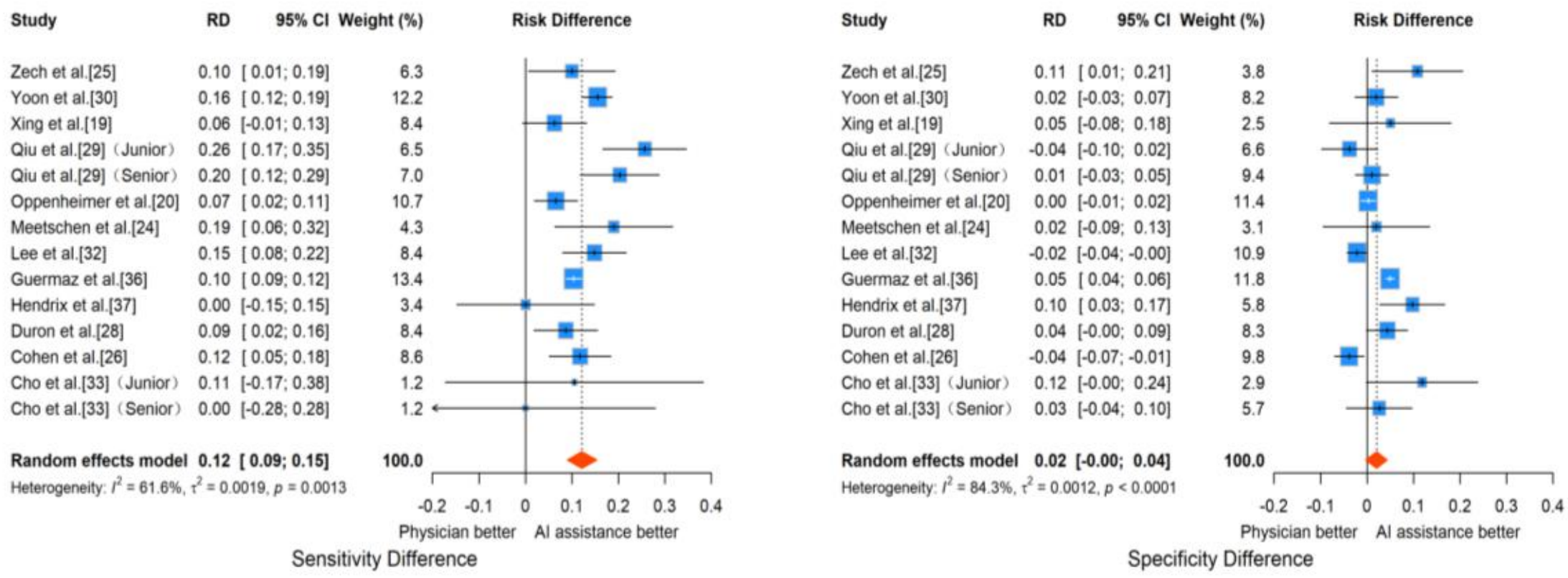

Figure S3. Forest Plots Illustrating the Effect of AI Assistance on Sensitivity and Specificity for X-Ray Imaging

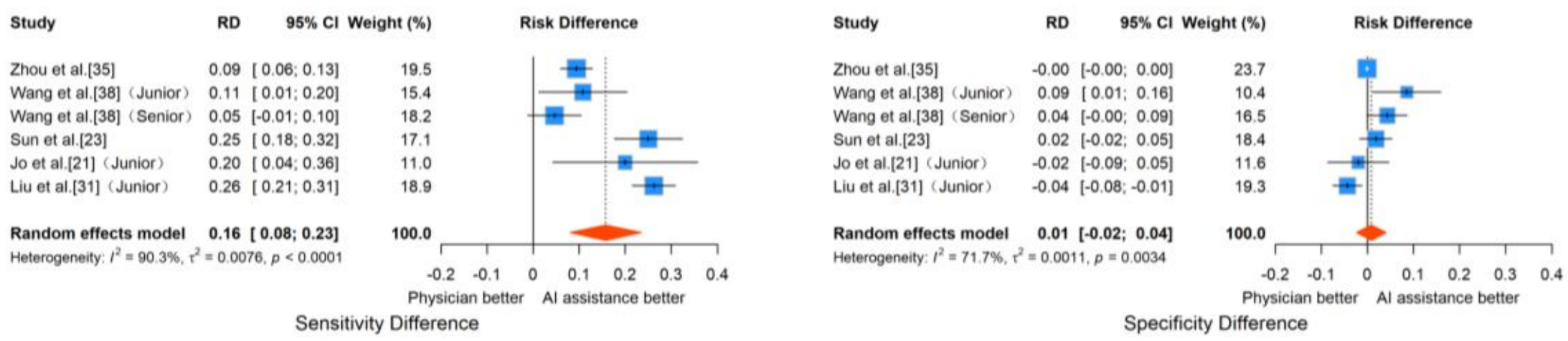

Figure S4. Forest Plots Illustrating the Effect of AI Assistance on Sensitivity and Specificity for CT or MRI Modalities

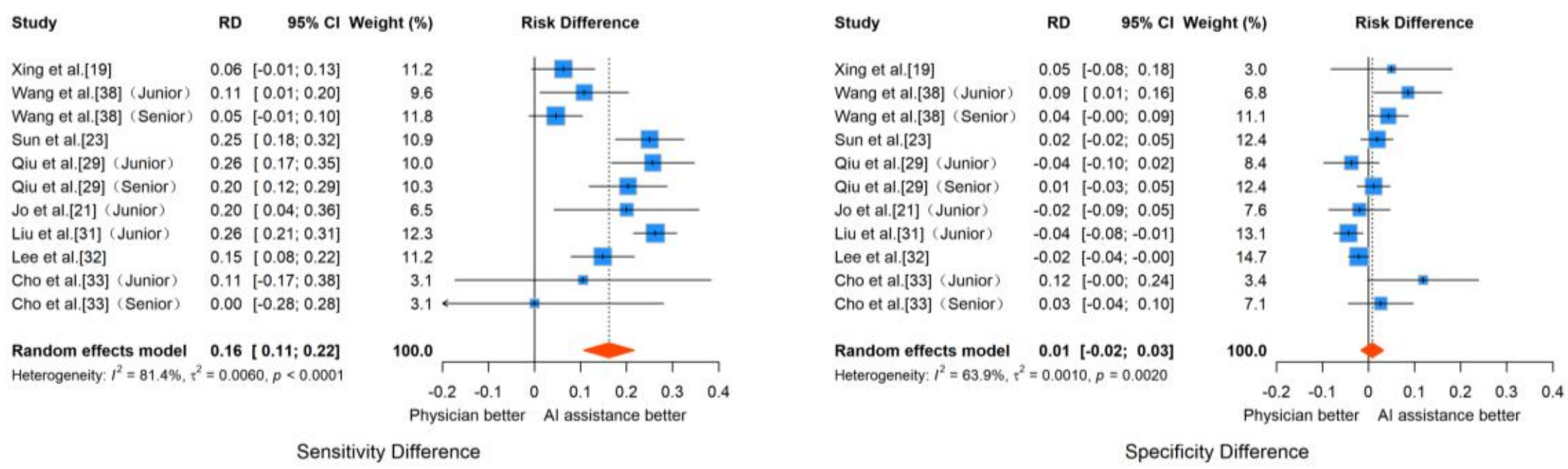

Figure S5. Forest Plots Illustrating the Effect of AI Assistance on Sensitivity and Specificity in Asian Cohorts

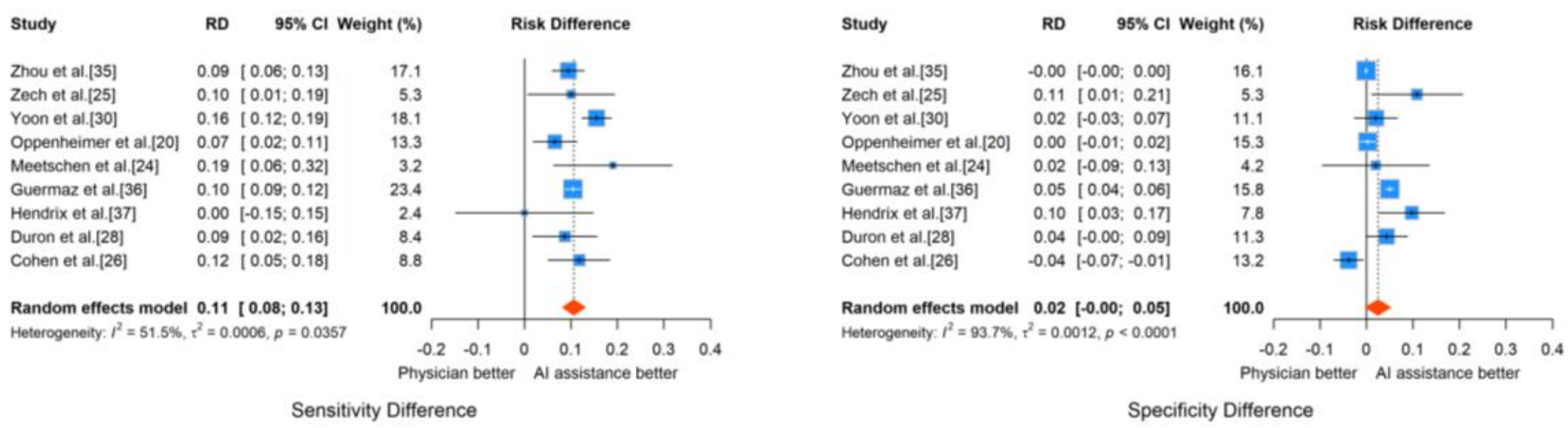

Figure S6. Forest Plots Illustrating the Effect of AI Assistance on Sensitivity and Specificity in European and American Cohorts

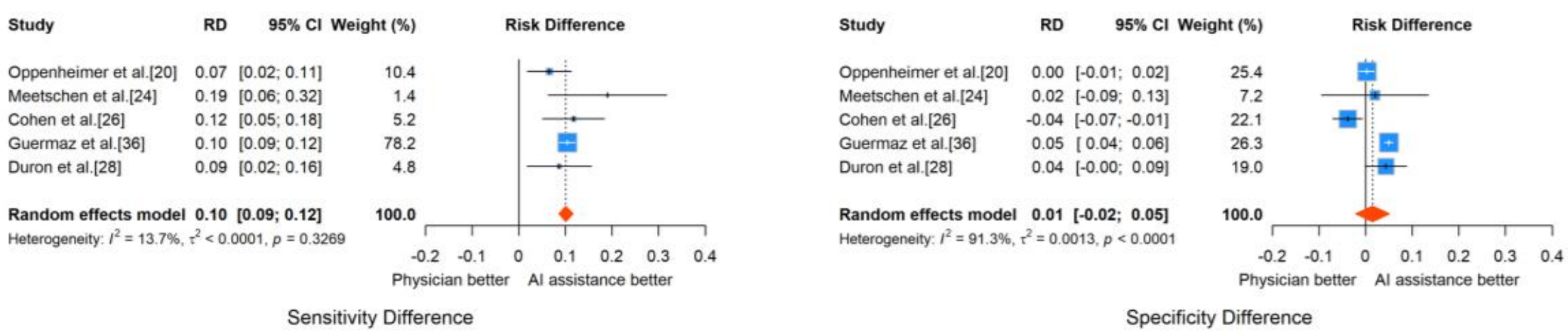

Figure S7. Forest Plots Illustrating the Effect of AI Assistance on Sensitivity and Specificity Using the Gleamer BoneView Algorithm

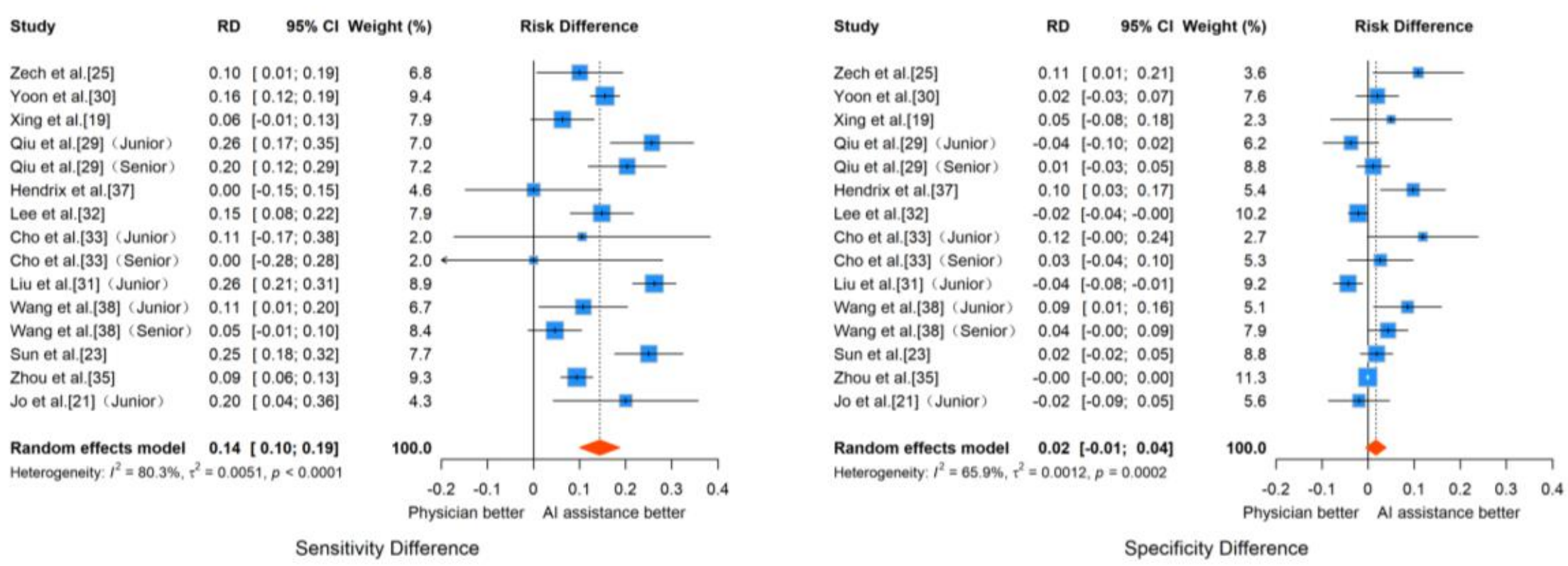

Figure S8. Forest Plots Illustrating the Effect of AI Assistance on Sensitivity and Specificity Using Other AI Algorithms

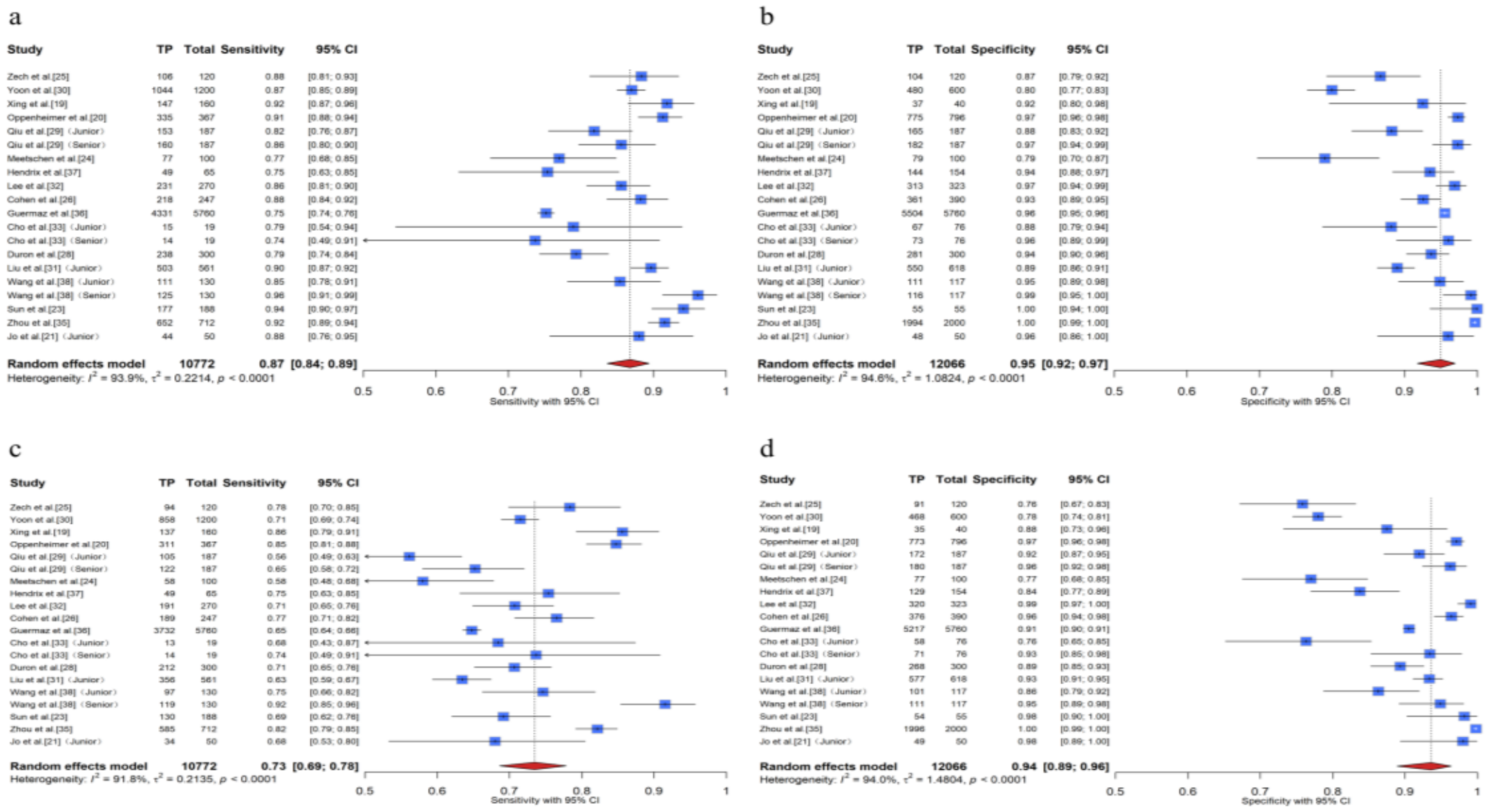

Figure S9. Comparison of Pooled Absolute Sensitivity and Specificity Between AI-Assisted and Independent Physician Diagnosis. (a) Pooled sensitivity for AI-assisted diagnosis; (b) Pooled specificity for AI-assisted diagnosis; (c) Pooled sensitivity for independent physician diagnosis; (d) Pooled specificity for independent physician diagnosis.

PRISMA 2020 Checklist

Section and Topic	Item #	Checklist item	Location where item is reported
**TITLE**			
Title	1	Identify the report as a systematic review.	Page 1, Line 1
**ABSTRACT**			
Abstract	2	See the PRISMA 2020 for Abstracts checklist.	Page 1, Lines 3–20
**INTRODUCTION**			
Rationale	3	Describe the rationale for the review in the context of existing knowledge.	Page 1, Lines 22–45
Objectives	4	Provide an explicit statement of the objective(s) or question(s) the review addresses.	Page 1, Lines 46–52
**METHODS**			
Eligibility criteria	5	Specify the inclusion and exclusion criteria for the review and how studies were grouped for the syntheses.	Page 3, Lines 15–30
Information sources	6	Specify all databases, registers, websites, organisations, reference lists and other sources searched or consulted to identify studies. Specify the date when each source was last searched or consulted.	Page 3, Lines 11–14
Search strategy	7	Present the full search strategies for all databases, registers and websites, including any filters and limits used.	Page 3, Lines 12–14
Selection process	8	Specify the methods used to decide whether a study met the inclusion criteria of the review, including how many reviewers screened each record and each report retrieved, whether they worked independently, and if applicable, details of automation tools used in the process.	Page 3, Lines 11–13; Page 3, Lines 31–33
Data collection process	9	Specify the methods used to collect data from reports, including how many reviewers collected data from each report, whether they worked independently, any processes for obtaining or confirming data from study investigators, and if applicable, details of automation tools used in the process.	Page 3, Lines 34–36
Data items	10a	List and define all outcomes for which data were sought. Specify whether all results that were compatible with each outcome domain in each study were sought (e.g. for all measures, time points, analyses), and if not, the methods used to decide which results to collect.	Page 3, Lines 36–38; Page 4, Lines 1–10
	10b	List and define all other variables for which data were sought (e.g. participant and intervention characteristics, funding sources). Describe any assumptions made about any missing or unclear information.	Page 3, Lines 36–38
Study risk of bias assessment	11	Specify the methods used to assess risk of bias in the included studies, including details of the tool(s) used, how many reviewers assessed each study and whether they worked independently, and if applicable, details of automation tools used in the process.	Page 3, Lines 39–41
Effect measures	12	Specify for each outcome the effect measure(s) (e.g. risk ratio, mean difference) used in the synthesis or presentation of results.	Page 4, Lines 11–18
Synthesis	13a	Describe the processes used to decide which studies were eligible for each synthesis (e.g. tabulating the study intervention characteristics and	Page 4,

Section and Topic	Item #	Checklist item	Location where item is reported
methods		comparing against the planned groups for each synthesis (item #5)).	Lines 19–28
	13b	Describe any methods required to prepare the data for presentation or synthesis, such as handling of missing summary statistics, or data conversions.	Page 4, Lines 11–13
	13c	Describe any methods used to tabulate or visually display results of individual studies and syntheses.	Page 5–7
	13d	Describe any methods used to synthesize results and provide a rationale for the choice(s). If meta-analysis was performed, describe the model(s), method(s) to identify the presence and extent of statistical heterogeneity, and software package(s) used.	Page 4, Lines 11–18
	13e	Describe any methods used to explore possible causes of heterogeneity among study results (e.g. subgroup analysis, meta-regression).	Page 4, Lines 19–35; Page 7, Lines 1–15
	13f	Describe any sensitivity analyses conducted to assess robustness of the synthesized results.	NA
Reporting bias assessment	14	Describe any methods used to assess risk of bias due to missing results in a synthesis (arising from reporting biases).	Page 7, Lines 16–18
Certainty assessment	15	Describe any methods used to assess certainty (or confidence) in the body of evidence for an outcome.	NA
**RESULTS**			
Study selection	16a	Describe the results of the search and selection process, from the number of records identified in the search to the number of studies included in the review, ideally using a flow diagram.	Page 5, Lines 1–12
	16b	Cite studies that might appear to meet the inclusion criteria, but which were excluded, and explain why they were excluded.	Page 5, Lines 10–12
Study characteristics	17	Cite each included study and present its characteristics.	Page 5, Lines 13–20
Risk of bias in studies	18	Present assessments of risk of bias for each included study.	Page 5, Lines 21–28
Results of individual studies	19	For all outcomes, present, for each study: (a) summary statistics for each group (where appropriate) and (b) an effect estimate and its precision (e.g. confidence/credible interval), ideally using structured tables or plots.	Page 6, Lines 1–20
Results of syntheses	20a	For each synthesis, briefly summarise the characteristics and risk of bias among contributing studies.	Page 5–6
	20b	Present results of all statistical syntheses conducted. If meta-analysis was done, present for each the summary estimate and its precision (e.g. confidence/credible interval) and measures of statistical heterogeneity. If comparing groups, describe the direction of the effect.	Page 6, Lines 21–28
	20c	Present results of all investigations of possible causes of heterogeneity among study results.	Page 7, Lines 1–25
	20d	Present results of all sensitivity analyses conducted to assess the robustness of the synthesized results.	NA
Reporting biases	21	Present assessments of risk of bias due to missing results (arising from reporting biases) for each synthesis assessed.	Page 7, Lines 16–20
Certainty of	22	Present assessments of certainty (or confidence) in the body of evidence for each outcome assessed.	NA

Section and Topic	Item #	Checklist item	Location where item is reported
evidence			
**DISCUSSION**			
Discussion	23a	Provide a general interpretation of the results in the context of other evidence.	Page 8, Lines 1–30
	23b	Discuss any limitations of the evidence included in the review.	Page 9, Lines 1–20; Page 10, Lines 9–15
	23c	Discuss any limitations of the review processes used.	Page 10, Lines 1–8
	23d	Discuss implications of the results for practice, policy, and future research.	Page 9, Lines 1–20; Page 10, Lines 9–15
**OTHER INFORMATION**			
Registration and protocol	24a	Provide registration information for the review, including register name and registration number, or state that the review was not registered.	Page 1, Line 5; Page 3, Line 5
	24b	Indicate where the review protocol can be accessed, or state that a protocol was not prepared.	NA
	24c	Describe and explain any amendments to information provided at registration or in the protocol.	NA
Support	25	Describe sources of financial or non-financial support for the review, and the role of the funders or sponsors in the review.	Declarations
Competing interests	26	Declare any competing interests of review authors.	Declarations
Availability of data, code and other materials	27	Report which of the following are publicly available and where they can be found: template data collection forms; data extracted from included studies; data used for all analyses; analytic code; any other materials used in the review.	Declarations

From: Page MJ, McKenzie JE, Bossuyt PM, Boutron I, Hoffmann TC, Mulrow CD, et al. The PRISMA 2020 statement: an updated guideline for reporting systematic reviews. BMJ 2021;372:n71. doi: 10.1136/bmj.n71. This work is licensed under CC BY 4.0. To view a copy of this license, visit https://creativecommons.org/licenses/by/4.0/
